# Toward Greener Multilayer Packaging Material Solutions Based on Microbial Protein and Polyhydroxyalkanoate

**DOI:** 10.1021/acsaenm.5c01169

**Published:** 2026-02-05

**Authors:** Kiran Reddy Baddigam, Elodie Guilloud, Anna J. Svagan, Bor Shin Chee, Buket Alkan Tas, Margaret Brennan Fournet, Kim Windey, Maria Batista, Cristiana A. V. Torres, Filomena Freitas, Mikael S. Hedenqvist

**Affiliations:** † Department of Fibre and Polymer Technology, Polymeric Materials Division, School of Engineering Sciences in Chemistry, Biotechnology and Health. KTH Royal Institute of Technology, 100 44 Stockholm, SE-100 44 Stockholm, Sweden; ‡ TUS Technological University of the Shannon, Centre for Polymer Sustainability, PRISM Research Institute, Midlands Midwest, Westmeath N37 HD68, Ireland; § Avecom Nv, Wondelgem, Ghent 9032, Belgium; ∥ Valpromic Nv, Wondelgem, Ghent 9032, Belgium; ⊥ UCIBIO − Applied Molecular Biosciences Unit, School of Science and Technology, NOVAUniversity Lisbon, Caparica 2829-516, Portugal; # Associate Laboratory I4HB - Institute for Health and Bioeconomy, School of Science and Technology, NOVA University Lisbon, Caparica 2829-516, Portugal

**Keywords:** microbial protein, single-cell protein, polyhydroxyalkanoate, laminate, packaging

## Abstract

Plasticized microbial (single cell) proteins (MPs) can be used to produce ductile and flexible plastic films with good oxygen barrier properties. However, as with other hydrogen-bond-forming oxygen barrier materials, like ethylene–vinyl alcohol copolymer (EVOH), they need to be protected from moisture because moisture decreases the oxygen barrier properties. Here, we solved the problem by producing three-layer laminate films that are fully biobased and biodegradable. Two different MP films (originating from a mixed microbial culture and *Delftia tsuruhatensis* biomass) were sandwiched between two different moisture-shielding polyhydroxyalkanoate (PHA) films (a poly­(3-hydroxybutyrate-*co*-3-hydroxyvalerate) and a poly­(3-hydroxybutyrate-*co*-3-hydroxyhexanoate) material). The low-temperature melting features of the PHAs made them suitable for lamination through hot-pressing with the MPs. Liquid-water-resistant and UV-blocking laminates could be obtained, where the individual layers were also possible to delaminate as a possible recycling solution, where the MP layer could potentially be used as a fertilizer and the PHA mechanically recycled into similar or other products or composted. The laminates showed, in the best cases, an oxygen permeability of 2 cm^3^ mm/(m^2^ day atm) and a water vapor permeability below 0.1 g mm/(m^2^ day). All in all, the concept is promising as a sustainable biobased alternative to today’s fossil-based EVOH-laminate packaging solutions.

## Graphical abstract

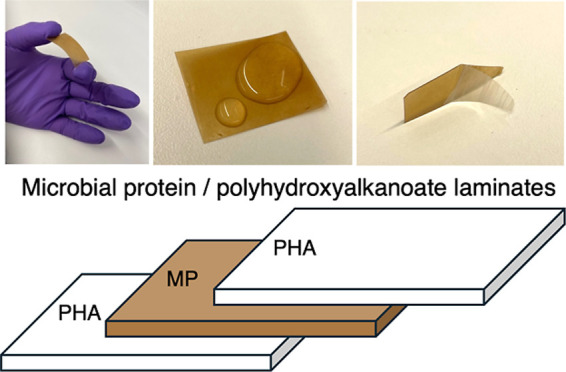

## 1 Introduction

There is a need to reduce the negative environmental impact of plastics. The incineration of plastics leads to the generation of CO_2_ and global warming, and the effects of global warming lead to more intense, longer, and more frequent heatwaves.^1–3^ This, in turn, leads to more frequent failure of heat-sensitive plastic products and consequently increased production of plastics and, in the end, more material going for incineration.^4^ It is easy to see that this is a vigorous self-strengthening circle.^5^ A way of breaking this circle is to use industrial composting for the end of life of the plastic products, which evidently requires biodegradable polymers.^6–8^ If the polymer is obtained from biobased sources, the need for fossil oil to produce plastics will be less and the carbon footprint thus lowered. If the biobased resource comes as a byproduct or side-stream from an existing or future industrial process, the solution may be even more sustainable. Another issue is the increasing plastic litter in both marine habitats and on land. Most polymers are quite chemically persistent in these environments. However, erosion and wear lead to the formation of nano- and microplastics,^9^ which present risks and potential issues for the environment, plants, animal life, and human health.^10–12^ Mechanical, chemical, and microbial enzymatic catalysis recycling are yet other possible ways of reducing environmental threats and facilitating more efficient use of the global resources.^13,14^

Packaging materials are of greatest concern because of their normally short service life, generating pollution much faster than materials in, e.g., automotives and built construction. Packaging is often also composed of several layers of materials, which makes recycling more complicated, especially when different types of materials are combined.^15–19^ Ethylene–vinyl alcohol copolymer (EVOH) is today the most commonly used gas-barrier polymer in food packaging. Its moisture sensitivity means that often it has to be laminated with hydrophobic polymers, such as polyethylene and polypropylene (the latter if, e.g., hot-filling is needed).^20^ Tie layers are also needed between EVOH and the outer layers, resulting in many food packagings having at least five layers. EVOH is an expensive fossil-oil-based nonbiodegradable polymer, and there is a need to replace it with biobased alternatives. This is, however, a challenging task, because the options of alternative existing or potential high-barrier materials, biobased materials, are limited.^21^

Microbial “single-cell” protein (MP/SCP) has been shown to have promising packaging-related properties,^22^ including oxygen barrier properties,^23^ which makes it a potential competitive alternative to EVOH. As with EVOH, it must be laminated with a more hydrophobic coating for applications in moist environments. Polyhydroxyalkanoate (PHA) is here a viable option. By combining it with MP, the multilayer packaging can be produced mainly, if not fully, from microbial biobased materials. These microbes can be cultivated from, e.g., food waste and, indeed, be regenerated from using the post-use packaging as a feedstock. Apart from using potentially sustainable sources, this packaging is readily biodegradable and produces less persistent microplastics. In fact, MPs, as with other proteins, work as a fertilizer and nutrition for plants and animals^24^ and can be converted to almost 100% biogas in anaerobic conditions,^25^ and PHAs are biodegradable in many types of environments.^26^

In this work, we explore for the first time the potential of multilayered films of microbial protein and PHA as future packaging material options, in line with previous work on polylactide (PLA)-laminated wheat gluten films,^27,28^ as well as PLA-laminated potato fruit juice films.^29^ We have shown that MP from a mixed microbiome, fed on potato peel, has very low oxygen permeability, even at 50% relative humidity.^23^ Besides this MP (we refer to it here as M-MP; the first M refers to a mixed microbiome), we are also assessing the potential of using MP produced by a pure culture of *Delftia tsuruhatensis* (hereafter termed D-MP or Delftia-MP), an interesting microbiome that can feed on terephthalic acid from depolymerized poly­(ethylene terephthalate) (PET, even metallized). Terephthalic acid is the monomer in PET that is the most difficult to recycle by microbes. Hence, if it is possible to use Delftia MP for new plastics, PET recycling can take a fully microbial route. Microbial proteins (single-cell proteins) are either referred to as dried microbial cells (bacteria, microalgae, yeasts, and fungi) or the extracted protein-rich material from these (besides the proteins, polysaccharides, fats, and minerals are usually present). They are produced in a biorefinery using a wide variety of substrates/sources, such as low-cost waste streams based on, e.g., sugar-industry waste, orange pulp, and coffee husk.^30^ The unextracted single-cell material can be made into cohesive films with the addition of a plasticizer (e.g., future packaging applications), but the cohesivity of the films depends on the level of disintegration of the cells; a more disintegrated cell system becomes more homogeneous with better material cohesivity.^23,25^

Two different PHAs were tested, which have a sufficiently low melting region to be able to laminate these with the microbial proteins: the copolymer poly­(3-hydroxybutyrate-*co*-3-hydroxyvalerate) (PHBV) with 19 mol % 3-hydroxyvalerate and the copolymer poly­(3-hydroxybutyrate-*co*-3-hydroxyhexanoate) (PHBH) with 10 mol % 3-hydroxyhexanoate content. The physicochemical properties of the individual materials as well as the multilayers of MP (with glycerol as plasticizer) and PHA were here assessed, including also oxygen and water vapor barrier properties.

## 2 Experimental Section

### 2.1 Materials

The spray-dried MP powder with a protein content of 68%, obtained by cultivation of a mixed microbiome with potato peel as the feedstock (M-MP), was provided by Valpromic nv (Belgium).^23^ Spray-dried Delftia-MP, or D-MP, with a protein content of 80%, provided by Avecom nv, was produced by cultivation of *D. tsuruhatensis* fed with chemically depolymerized PET.^31–33^ *D. tsuruhatensis* cells had a PHA content of 3 wt %, comprising a PHBV with 5% 3-hydroxyvalerate (3HV). PHBV with a 3HV content of 19 mol % was produced by a mixed microbiome using fermented cheese whey as feedstock.^34^ It was recovered from the freeze–dried biomass with chloroform by Soxhlet extraction, followed by purification by precipitation in ice-cold ethanol, as described by Pereira et al.^35^ The poly­(3-hydroxybutyrate-*co*-3-hydroxyhexanoate) (PHBH) with 10 mol % 3-hydroxyhexanoate (HH) (Bluepha PHA BP350-05 (Bluepha (China)) was purchased from Helian Polymers (The Netherlands). It was provided as 10 cm wide extruded tape.

### 2.2 Film Preparation

To obtain flexible films, the MP powders were mixed with glycerol in a mortar with a pestle for 5 min and then placed in a desiccator for about 3 h before pressing. The M-MP mixture was placed in a circular steel frame/mold with a thickness of 100 μm and a diameter of 3 cm and with antisticking PET sheets on both sides, with also two steel plates below and above the PET sheets. The whole assembly was placed in a Fontijne Press (The Netherlands) and compression molded. The optimal pressing conditions were determined to be 5 min pressing at 110 °C with a force of 60 kN. The films were removed from the PET sheets and the press after cooling. Because of the limited availability of Delftia material, the D-MP films were pressed in a Graveda Graspresso EPIC press (15 tons) (CRP Press Import—Export, Germany) using 1 MPa (determined to be sufficient pressure to produce films). Films with a glycerol content of 15 to 40 wt % were pressed initially. From here on, “wt %” is expressed as “%”. It turned out that a glycerol content of at least 25% was necessary to obtain sufficiently flexible films in the case of D-MP, and 40% yielded, as expected, the most flexible films. Since the oxygen barrier properties decrease with increasing glycerol content, it was decided to go further with D-MP films having 25 and 30% glycerol (D-MP-25 and D-MP-30). For M-MP, it turned out that also the use of 20% glycerol yielded sufficiently flexible films; hence, 20, 25, and 30% glycerol films were considered further for this material (M-MP-20, M-MP-25, and M-MP-30). For tensile testing, a rectangular 5 × 12 cm mold with a thickness of 100 μm was used, and films were pressed in the Fontijne Press as described above.

PHBV powder and PHBH (pieces cut out from the purchased extruded film product) were compression molded in the Graveda Graspresso EPIC press using a 40 μm thick steel mold with a diameter of 3 cm with PTFE films as antisticking sheets. It turned out that good-quality films could be made by pressing the material at 110 °C, using 1 MPa of pressure for 5 min. For mechanical testing, a 100 μm thick rectangular (5 × 12 cm^2^) steel mold/frame was used, and these samples were pressed in the Fontijne Press at 60 kN. It should be pointed out the final MP and PHA films were mostly thicker, to different extents, than the thickness of the molds used, depending on their ability to float out under the pressing operation.

### 2.3 Lamination

A large set of tests were performed to laminate MP with PHA layers on each side (PHA/MP/PHA), using the Graveda Graspresso EPIC press, to have better control of the pressing procedure. Continuous fracture-free laminates which had a layer cohesion sufficient for flexing the laminate were obtained with combinations of PHBV or PHBH with Delftia-MP (25 and 30% glycerol, referred to as D-MP-25/PHBV, D-MP-30/PHBV, D-MP-25/PHBH, and D-MP-30/PHBH) or M-MP (20 and 25% glycerol, referred to as M-MP-20/PHBV, M-MP-25/PHBV, M-MP-20/PHBH, and M-MP-25/PHBH). M-MP with 30% glycerol was not evaluated in a laminate structure since, again, a higher than necessary glycerol content deteriorates the gas barrier properties. Further details on the lamination procedure are described below in the Results and Discussion section.

### 2.4 Characterization

#### 2.4.1 Differential Scanning Calorimetry (DSC)

The DSC measurements were carried out using the following method: stabilization at −30 °C for 10 min, first heating to 220 °C at a rate of 20 °C/min, equilibration at 220 °C for 10 min, cooling from 220 to 30 °C at 20 °C/min, −30 °C for 10 min, and finally a second heating to 220 °C. The 10 mg samples were placed in aluminum pans with a hole to allow flushing with nitrogen using a gas flow of 50 mL/min.

#### 2.4.2 Fourier Transform Infrared Spectroscopy (FTIR)

FTIR spectroscopy analyses of the films were performed using a PerkinElmer Spectrum 100 FT-IR spectrometer in attenuated total reflection mode. Spectra were recorded from 600 to 4000 cm^–1^ (32 scans and a resolution of 4 cm^–1^). The films were placed in a vacuum desiccator for a minimum of 48 h prior to measurements. The amide I region (1700–1580 cm^–1^) was deconvoluted using the Multiple Peak Fit function (iteration algorithm: Levenberg–Marquardt) in Origin 2020. Prior to fitting, the amide I region (1700–1580 cm^–1^) was first baseline-corrected. The positions of the centers of the Gaussian-shaped peaks were fixed during optimization.

#### 2.4.3 Tensile Testing

Tensile tests were carried out using an Instron 5944 universal testing machine. Rectangular samples, 5 cm long and 1 cm wide, were cut out from the pressed films and then placed in a conditioning room for 2 days (23 °C and 50% relative humidity (RH)) before testing. The thickness was then measured with a micrometer at 3 points, and the average value was taken. Analyses were performed with a preload of 0.02 N, a strain rate of 10 mm/min, and an initial clamp-to-clamp distance of 2 cm. Up to six replicates were used for each material.

#### 2.4.4 Dynamic Mechanical Analysis (DMA)

DMA analyses were performed using a multifrequency strain method. Rectangular specimens, 2 cm long and 0.8 cm wide, were cut from the pressed PHBV and PHBH films. Testing was performed after drying in a desiccator for at least 2 days. The conditions used were a preload force of 0.01 N and a strain amplitude of 0.1%. Each sample was conditioned for 5 min at −30 °C before the heating ramp started (with 3 °C/min to 80 °C).

#### 2.4.5 Oxygen Permeation

The oxygen permeation properties were obtained using PermeO_2_ (ExtraSolution, Italy) following the ASTM D3985 standard. Measurements were performed using 99.9995% specialty grade oxygen at 50% RH, 23 °C, and 1 bar (partial pressure difference). The films were masked with aluminum foil with a 1.6 cm diameter hole (area 2.01 cm^2^). The samples were tested for up to 24 h. A preconditioning was performed at 50% RH and 23 °C in nitrogen before the measurements.

#### 2.4.6 Water Vapor Transmission Rate (WVTR)

The water vapor permeation properties were determined using a PermeH2O instrument (ExtraSolution, Italy). Similar to the oxygen permeability test, the films were tightly sandwiched with aluminum masks and loaded into a diffusion cell for conditioning. The samples were tested at 38 °C with a 90%—0% RH gradient for up to 24 h. Also here, a preconditioning was performed at 38 °C in dry nitrogen before the measurements.

#### 2.4.7 UV–Visible (UV–Vis) Spectrophotometry

UV–vis spectrophotometry was performed using a UV–vis spectrophotometer (Shimadzu UV 2550, Japan). The total transmittance measurements were conducted by utilizing the ISR-2200 integrating sphere and BaSO_4_ as the standard white reference.

## 3 Results and Discussion

In order to produce the laminates and decide on the pressing temperatures, the thermal properties of each material were investigated with calorimetry as well as the thermomechanical (PHA materials) and tensile properties of the individual materials. Then, the protein secondary structures were presented, which was followed by a description of the general/optical and UV–vis properties of individual films and laminates. Finally, the oxygen and water-vapor permeances of the individual films and laminates were presented, involving both types of PHA and both types of microbial proteins.

### 3.1 Calorimetry

As described in the Experimental Section, a pressing temperature of 110 °C was found to be the most suitable for producing films of both material types (MP and PHA). The first heating of M-MP with different glycerol contents revealed mainly a broad endothermal peak, peaking at 131–139 °C (Figure 1a). This was due to the evaporation of mainly water. Notice the larger peak (evaporation energy went from 23 J/g (20% glycerol) to 39 J/g (30%)) and the higher peak temperature with higher glycerol content present. This indicated a larger amount of water present (attracted by glycerol), as well as the larger amount of glycerol present. This evaporation peak made it impossible to determine any glass transition of the films. The slight upturn at a temperature above the main peak was due to partial evaporation of glycerol. The second heating, without the evaporated water, made it possible to estimate the glass transition temperature region, which increased with the initial glycerol content, revealing that a sizable amount of glycerol was left in the sample after the first heating (Figure 1b). Hence, the estimated glass-transition region (20–100 °C) from the second heating is an overestimate; nevertheless, it shows that going for ≥100 °C in the pressing operation will result in a softened/malleable film that will adhere better to the PHA layers. Zhang et al.^36^ reported, from DSC data, a glass transition region that occurred between 30 and 65 °C for a soy protein material with 23% glycerol at a quite low moisture content (2.8%). Hence, the glass transition regions reported here are similar to that of the soy system, indicating that the major effect dictating the glass transition temperature is the presence of plasticizer rather than the actual protein chemistry/structure (although both proteins are most likely intrinsically disordered systems).

**Figure 1:**
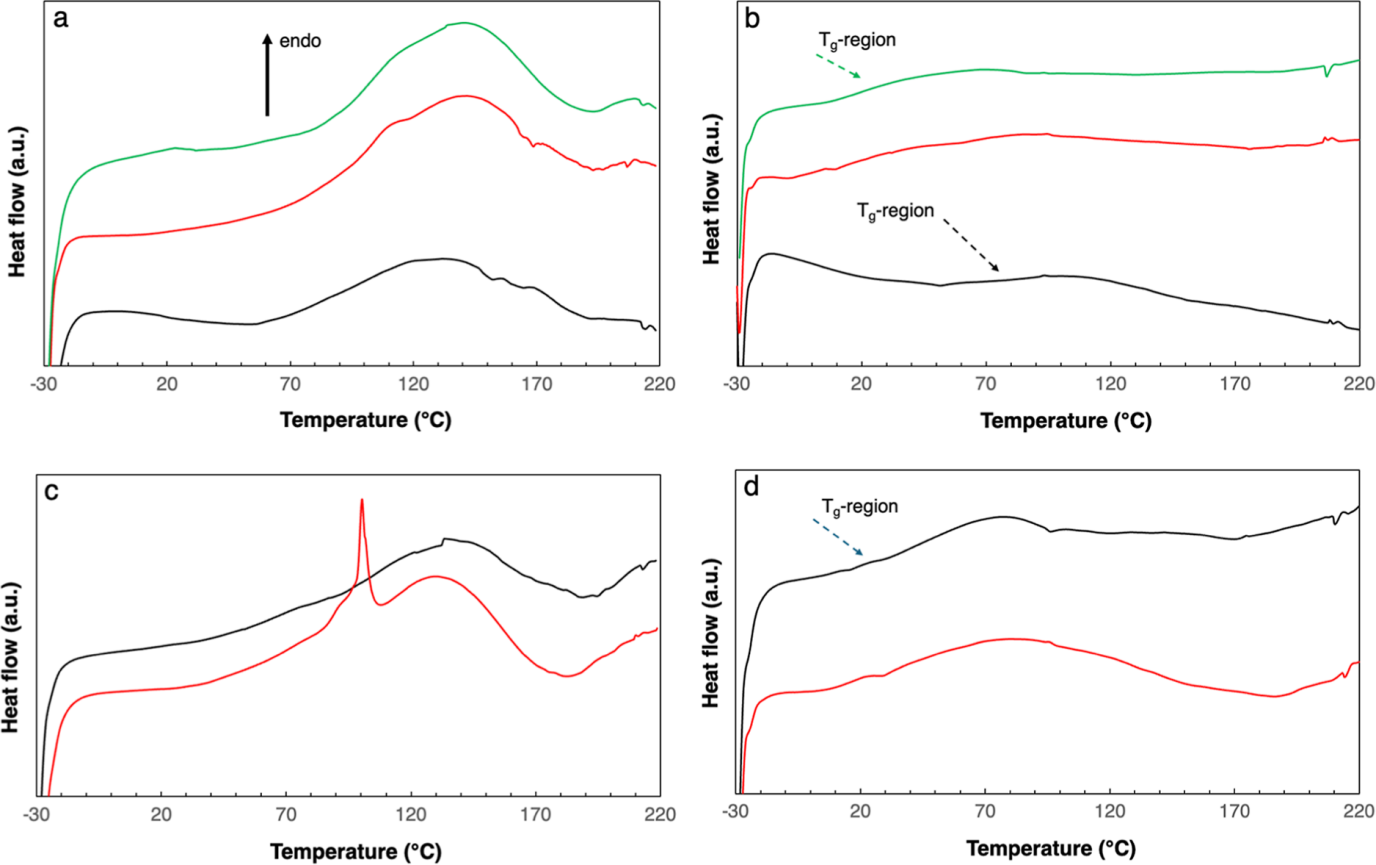
DSC curves representing M-MP films with 20 (black curve), 25 (red), and 30 (green) % glycerol: (a) first heating; (b) second heating. DSC curves representing D-MP films with 25 (black curve) and 30 (red) % glycerol: (c) first heating; (d) second heating. Arrows point to the middle of the glass transition region.

Similar to M-MP, the DSC curves of the first heating of the D-MP (Figure 1c) were characterized by a broad endotherm due to the evaporation of mainly water, peaking at ca. 130 °C, and the evaporation of glycerol at higher temperatures (observed as an upturn in the DSC curves above 175 °C). In addition, the sharp peak at 100 °C in the 30% glycerol sample indicated the presence of free phase-separated water in the sample. It also indicates that the broad endotherms in both MPs were due to water bonded in different ways to the protein/glycerol. The second heating also verified that there was a substantial amount of glycerol left in the sample after the first heating (Figure 1d). The glass-transition region stretched from around 10–60 °C. As for M-MP, it showed that using a pressing temperature above 100 °C ensures a soft MP more suitable for the lamination with the PHAs.

The DSC curves of the PHA materials showed complex melting patterns (Figure 2). The PHBV raw material showed a glass-transition temperature of 7 °C, and the melting occurred over a large interval, spanning from 50 to over 150 °C, with a maximum melting peak temperature of 150 °C (Figure 2a). The PHBH raw material showed an almost similar glass-transition temperature as the PHBV (5 °C) and also a broad melting in the similar range as the PHBV (Figure 2b). At least three different *T*_m_’s have been identified in the case of PHBH, which are associated with the melting of different types of lamellae.^37^ The first endotherm (*T*_mI_ ≈ 75 °C) corresponds to the melting of secondary lamellae, while the second endotherm (*T*_mII_ ≈ 110 °C) is attributed to the melting of primary lamellae. The endotherm at a higher temperature (*T*_mIII_ ≈ 130 °C) originates from the reorganization or thickening of both primary and secondary lamellae. There is a fourth shoulder at ca. 140 °C, which is either due to the existence of thicker lamellae or the reorganization into thicker lamellae of thinner lamellae. *T*_mI_ may also contain an endotherm that is due to the storage of the material at ambient conditions.^37^ Since the pressing of the laminates should be performed at a temperature suitable for both the MP and the PHA, it was decided to try pressing the PHAs at a temperature similar to that used for the MPs. After several tests with different temperatures and pressing times, it was observed that 110 °C was a good pressing temperature also for the PHAs. The DSC curves of these pressed films are also shown in Figure 2. The curves are relatively similar to those of the raw materials.

**Figure 2:**
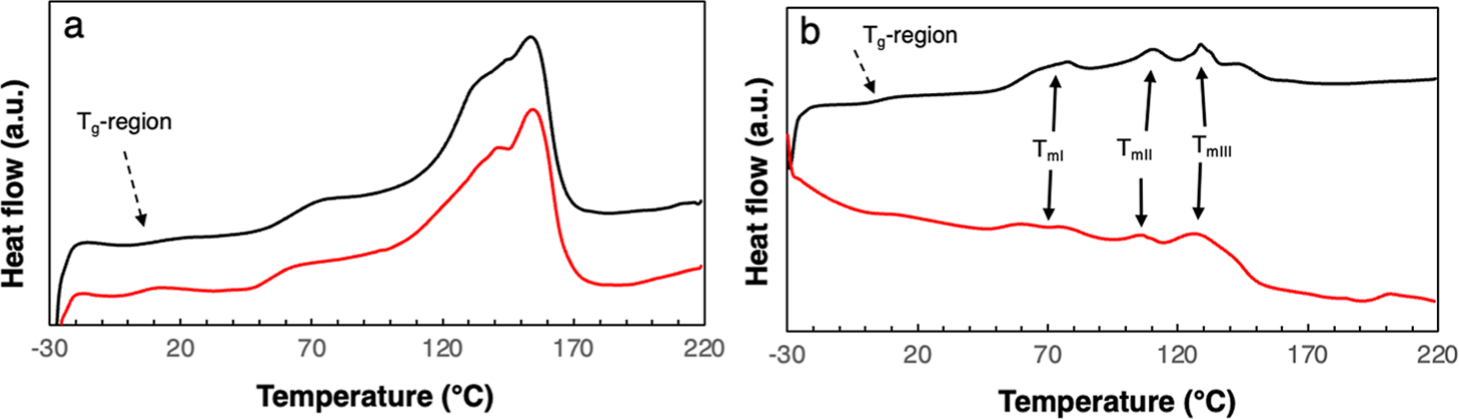
DSC heating curves of the (a) PHBV raw material (black curve) and pressed film (red) and (b) corresponding curves for PHBH.

### 3.2 Mechanical Properties

DMA was used to further characterize the PHA films (Figure 3). However, it was not possible to obtain reliable DMA data on the MPs. The glass-transition temperatures were somewhat lower than those obtained by DSC (4 °C for PHBV and 1 °C for PHBH, from the loss modulus), which is understandable, considering the different heating rates in the two methods. The constant decrease in the storage modulus, from the glassy state through the glass transition region and beyond up to the maximum temperature used (80 °C), was in agreement with the early and broad melting of these materials (above ca. 50 °C) (Figure 2).

**Figure 3:**
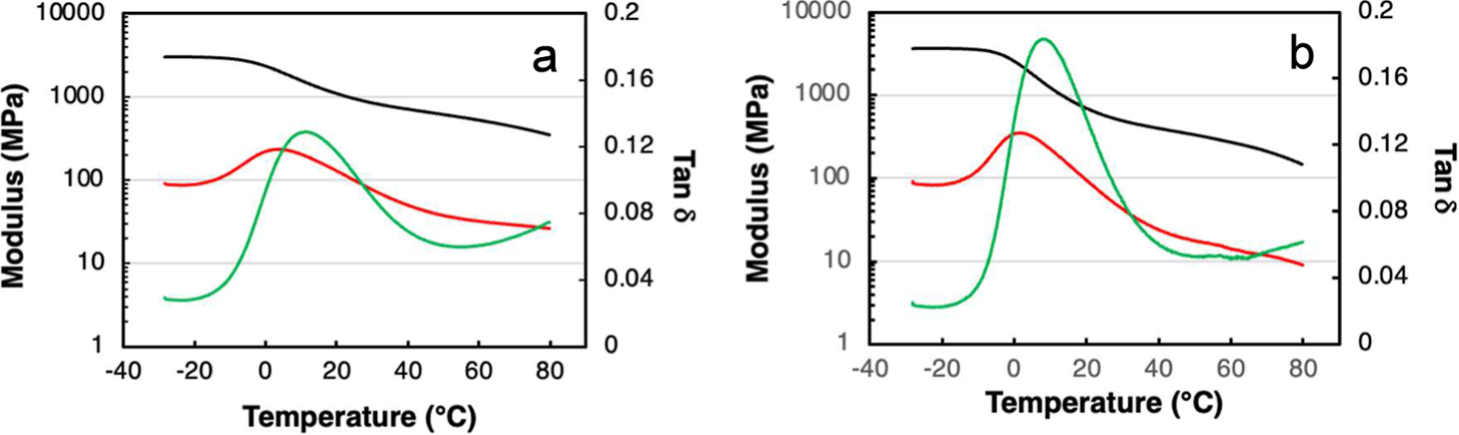
DMA curves of (a) PHBV and (b) PHBH. Black, red, and green curves refer to, respectively, the storage modulus, loss modulus, and damping (tan δ).

Before lamination, the tensile mechanical properties of the individual films were measured. Table 1 summarizes the mechanical results, and typical stress–strain curves are presented in Figure 4. As expected, the strength and stiffness were lower, and the ductility (strain at break) was higher for the M-MP material at higher glycerol content. The strain at break was also significantly higher than that of the material based on the Delftia strain. In fact, to obtain a D-MP film with high flexibility, 30% glycerol was needed. The D-MP-30 sample had a strain at break of only 4%, as compared to the over ≥50% value for M-MP (with less glycerol). The break was also more sudden/brittle-like for the D-MP material than for the material based on the mixed culture (M-MP); also, the strain at maximum stress was significantly lower than the strain at break for the latter material (Table 1). To summarize, the M-MP ductility outperformed that of the D-MP. The stiffness of the MPs here was significantly higher than those of the microbial protein obtained from a whey source (∼6–11 MPa, with 30% glycerol).^25^ The strength (maximum stress) was also significantly higher here than for the whey-based microbial protein (∼0.15–0.3 MPa), and the ductility (strain at break) of the whey-based MP was similar to that of D-MP-30 here. Hence, the cohesion of the MP films produced here was, in general, better than that of those made from whey. In fact, the present materials mechanically outperformed the MPs of similar origin (mixed microbiome) presented earlier.^22^

**Table 1:** Tensile Properties of the Films^a^,^b^

sample	maximum stress (MPa)	modulus (MPa)	strain at max. stress (%)	strain at break (%)
M-MP-20	2.5 ± 0.5	270 ± 49	42 ± 10	50 ± 11
M-MP-25	1.9 ± 0.1	65 ± 8	47 ± 5	67 ± 8
D-MP-30	3.5 ± 0.1	317 ± 76	3.8 ± 1.3	3.9 ± 1.2
PHBV	13.5 ± 1.0	1318 ± 275	3.2 ± 1.0	20 ± 7
PHBH	16.8 ± 0.8	541 ± 66	8.7 ± 1.0	45 ± 4

a ± values refer to one standard deviation; sample thicknesses: ca. 100 to 450 μm.

b From ref 23.

**Figure 4:**
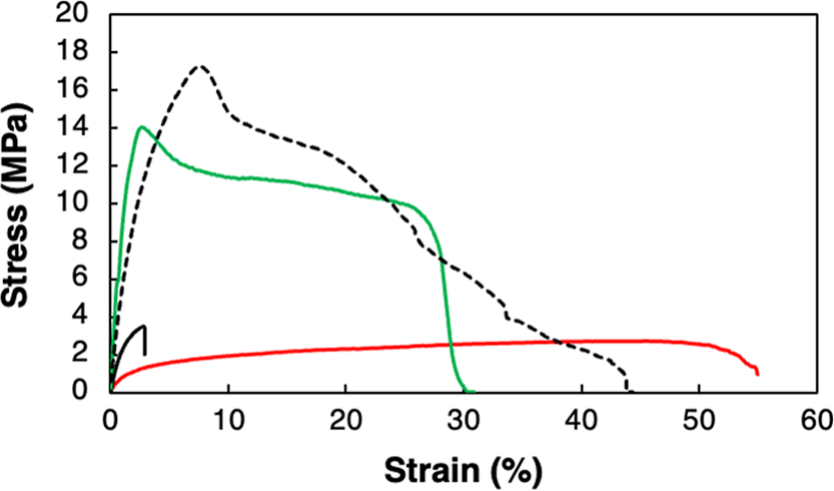
Examples of stress–strain curves for M-MP-20 (red curve), D-MP-30 (black solid curve), PHBV (green curve), and PHBH (broken curve).

The mechanical properties of PHAs depend on the type and content of comonomer, where an increase in comonomer content generally increases ductility and lowers the stiffness and strength. The relatively high ductility presented here (Figure 4 and Table 1) is expected based on the comonomer type and content.^38^

### 3.3 Protein Structure by FTIR

To explore if the difference in mechanical properties of the two microbial proteins was due to differences in the secondary structure of the proteins, the FTIR amide I region was analyzed in detail for the Delftia samples, which were then compared with that of the M-MP material reported in a previous study.^23^ The FTIR spectra of the Delftia materials are displayed in Figure S1 as well as the deconvoluted curves. The result of the deconvolution is presented in Table S1, and the values grouped into the four different types of secondary structure are given in Table 1, along with the M-MP values. The hot-pressed samples are expected to have a protein component that is more aggregated than that of the pristine material/powder. Indeed, the amount of β-sheets with strong hydrogen bonding, indicative of aggregation, was significantly higher in the pressed D-MP samples than in the powder. They were in a range similar to that observed for the pressed M-MP materials. However, the amount of β-turns was significantly higher in the M-MP samples, whereas the amount of α-helices and random coils and unordered structures was higher in the D-MP samples. This suggests a more folded structure in the M-MP protein; however, it is not evident how this would lead to a material with a significantly higher ductility, despite the lower glycerol content. It may depend on other factors also, including variations in the degree of mixing between the protein and glycerol and the difference in content of both protein and nonprotein components; neither D-MP nor M-MP are pure proteins.

### 3.4 Features and Appearance of the Individual Films and Laminates

Examples of the final films used in the lamination are shown in Figure 5. The PHA films were transparent, while the MP films were translucent. The D-MP films were less brown than the M-MP films at the same glycerol content (UV–vis data will be presented later).

**Figure 5:**
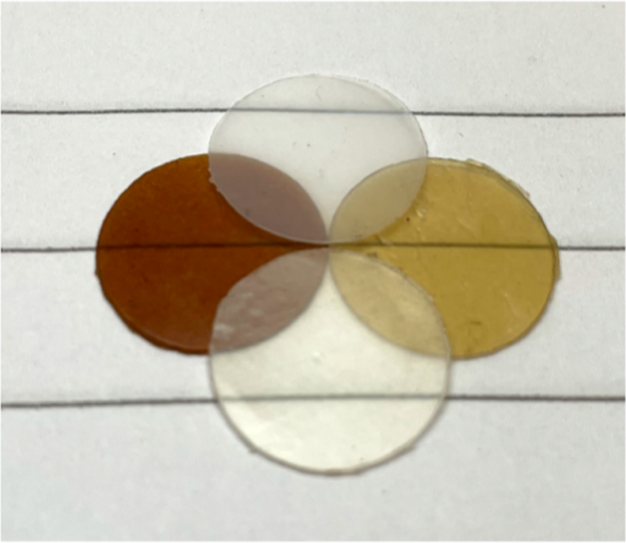
Films of M-MP-25 (left), D-MP-25 (right), PHBV (top), and PHBH (bottom), all pressed at 110 °C. These were punched out from pressed films and have a diameter of 11 mm.

In the lamination process, the temperature, pressure applied, and time of the pressing were varied. The main problems to overcome were to obtain good adhesion over the whole interface of the three layers and to ensure that the individual layers, specifically the middle MP layer, did not break. Temperatures tested were from 40 to 110 °C, and from these tests, it was observed that while the expansion of the middle layer decreased with lower temperature, which was beneficial to avoid cracking, the adhesion became poorer. The most important factor to consider turned out to be the size of the applied force/pressure. Also, it turned out to be important to use a small pressing time. Consequently, the best results were obtained by the use of a very low pressure for 30 s at 110 °C. The pressure was lower than the first reading (hence <1 MPa) of the press, simply enough to have a firm contact between and throughout the whole layers. Because of the elevated and soft material, some compression of the layers may still have occurred. It is important to state here that the lamination procedure adopted here was used to obtain laminates that could be evaluated without using a large amount of material (otherwise necessary in coextrusion). The materials used here were of limited amount, as is normally the case when new materials are developed. To assess fully the possibility of using this concept in food packaging and as a replacement for EVOH-based barrier solutions, it is important to upscale the MP/PHA systems and to evaluate these in a coextrusion/packaging film setup.

The laminate based on the three layers of M-MP-25/PHBV is displayed in Figure 6. The multilayer film was flexible enough to be bent, as shown in Figure 6a, and water droplets did not penetrate the laminate (Figure 6b, measured over ∼3 h, until the droplets had evaporated). The three layers could be easily disassembled, which can be beneficial in a recycling loop if the materials need to be in different recycling streams (Figure 6c). The PHAs can be thermally reprocessed into new products or go for biodegradation (they can be degraded through photooxidative, catalytic, thermal, and mechanical routes, and for complete breakdown in nature, degradation driven by microbes plays an important role).^38^ Illustration examples of the laminates used for the UV–vis and permeation experiments are given in Figure S2. It should also be noted that by lamination, any migration of glycerol from the MP layer is counteracted by the outer layers, as shown on wheat gluten laminated with polylactide.^27^ Even though the properties of the laminate are promising for future packaging applications, they are not as flexible as low-density polyethylene (LDPE)-based packagings, and in terms of rigidity, they would compare better with high-density polyethylene (HDPE), polypropylene, or PET-based packagings. Further optimization with regard to packaging parameters, besides coextrudability, needs optimization (e.g., sealability).

**Figure 6:**
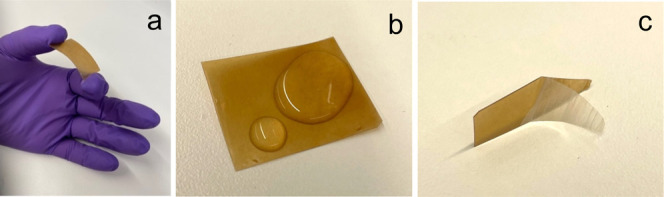
a) Illustration of the flexibility of the M-MP-25/PHBV 3-layer laminate, (b) water droplets placed on the laminate, and (c) illustration showing the separation of the different layers.

The total transmittance of some films and multilayers was determined to assess the transparency and UV-blocking effect of both pristine microbial protein films and when these were laminated with PHA layers. Typically, a polymer film that has a light transmission greater than 90% at 600 nm is transparent to the eye,^39^ and this was not the case for the MP films or the laminates. The films and multilayers analyzed were all translucent with a total transmittance of 55–75% at 800 nm (Figure 7). They should, however, not be compared individually since the thicknesses varied (ranging from around 150 to over 550 μm). However, noteworthy is that the UV transmittance (below 400 nm) was quite low for the Delftia-based samples and absent for the samples based on the mixed microbiome (M-MP). Hence, the MP-based films and multilayers are suitable in packagings for UV-sensitive food (e.g., milk products). As observed, the decrease in transmittance from the PHA layers was small or absent.

**Figure 7:**
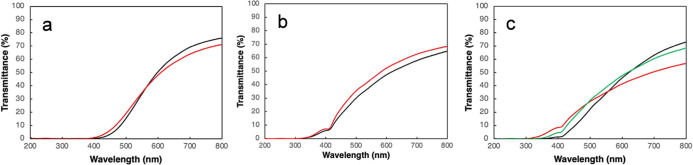
UV–vis total transmittance as a function of wavelength. (a) M-MP-20 (black curve) and M-MP-20/PHBH (red), (b) D-MP-25 (black) and D-MP-25/PHBH (red), and (c) D-MP-30 (black), D-MP-30/PHBV (red), and D-MP-30/PHBH (green).

### 3.5 Permeation Properties

The oxygen permeability (OP) increased with increasing glycerol content for the D-MP films, and the values were considerably higher than those of the M-MP films reported previously (Table 3).^23^ A possible explanation for this is the better packing of the M-MP protein molecules (refer to the β-turn content in Table 2). However, since these are not pure protein systems, other components in the material can also be responsible for the difference in the permeabilities. The MP films are too hygroscopic to be tested for water vapor transmission rate. The oxygen permeability of the two PHAs was both similar to that of the Delftia material with 25% glycerol (D-MP-25) (Table 3). The values are higher than that of a PHBV film with 3 mol % HV (∼3 cm^3^ mm/(m^2^ day atm)).^40^ The lower values of the PHBV with 3 mol % HV are expected, since the crystallinity is presumably higher than that of the present materials. It is also known that the way of processing these materials affects the crystallinity.^41^ The values of the PHBV and PHBH films here were similar to those of polylactide (PLA) and somewhat lower than that of poly­(butylene-*co*-adipate-*co*-terephthalate) (PBAT) (10–30 cm^3^ mm/(m^2^ day atm).^29^ Note that the permeation properties depend on the actual crystallinity of the different polyesters; therefore, differences can be expected. The specific (thickness normalized) water vapor transmission rate (sWVTR) of the two PHAs was similar (average values: 7–9 g mm/(m^2^ day)) (Table 3), which is significantly higher (and expected) than that reported for a PHBV with 3 mol % HV (∼0.2 g mm/(m^2^ day)) under a similar relative humidity gradient (85% RH), but at a lower temperature (23 °C rather than 38 °C).^42^ Besides the different temperatures, the differences in the values are again also expected due to the differences in the HV content. Shogren reported a 100% increase in WVTR when the content of HV increased from 6 to 18 mol %.^43^

**Table 2:** Secondary Structures (in %) Based on FTIR Analysis^a^

sample	D-powder	D-MP-25	D-MP-30	M-MP-20	M-MP-25
β-Turns	9.6	8.5	8.2	30.9	26.8
α-Helices and random coils/unordered	25.2	31.1	33.5	11.0	12.1
β-Sheets, weakly hydrogen-bonded peptide groups	43.9	9.7	11.6	15.6	11.8
β-Sheets, strongly hydrogen-bonded peptide groups	21.2	50.7	46.6	42.5	49.3

a Values on M-MPs from Reddy Baddigam.^23^

**Table 3:** Oxygen and Water Vapor Permeance

sample	thickness^a^	OTR^b^	OP^c^	WVTR^d^	sWVTR^e^
D-MP-25	330	54	18	-	-
D-MP-30	320	100	32	-	-
M-MP-25 ^23^	100–300		0.33 ± 0.7	-	-
PHBH	107–110	209 ± 49	15 ± 6	82 ± 16	9.2 ± 1.6
PHBV	98–109	268 ± 236	18 ± 24	68 ± 39	7.4 ± 4.3
D-MP-25/PHBH	335	47	16	16/>39^i^	5.4/>13^i^
D-MP-30/PHBH	422	>3121^f^	>1335^f^	>5.0	>2.2
D-MP-25/PHBV	554	>6.0^f^	>3.4^f^	0.26^h^	0.14^h^
D-MP-30/PHBV	759	18^g^	14^g^	0.04^h^	0.03^h^
M-MP-20/PHBH	302	6.0	1.8	>4.4^f^	>1.3^f^
M-MP-25/PHBH	405	23	9.5	19^g^	7.7^g^
M-MP-20/PHBV	657	3.1^h^	2.1^h^	0.3^h^	0.2^h^
M-MP-25/PHBV	368	14	5.2	6.2^g^	2.3^g^
M-MP-25/PHBV	256	19	5	2.0/>3.6^j^	0.5/>0.9^j^

a Film thickness (μm).

b Oxygen transmittance rate (cm^3^/(m^2^ day)).

c Oxygen permeability/permeance (cm^3^ mm/(m^2^ day atm)).

d Water vapor transmittance rate (g/(m^2^ day)).

e Specific water vapor transmittance rate (g mm/(m^2^ day)).

f Final value not reached within the experimental time (≤24 h).

g Final value not reached within the experimental time (≤24 h), extrapolated to steady state.

h Maximum value.

i Three-stage curve: the first value is based on the first part, and the final value is from the third stage, not reached within 24 h.

j Two-stage curve: the first value is based on the first part, and the final value is not reached within 24 h for the second part.

As expected, due to the similarity in OP for the different layers, the resulting oxygen permeances for the D-MP/PHA laminates were essentially the same as for the individual layers, although the values of the two samples were not conclusive, possibly due to voiding in some or all layers. For the sWVTR, the laminates with D-MP and PHBV showed an improvement compared to the PHBV film; hence, the MP material contributed to an increased water barrier, as it is exposed to low water uptake because of the surrounding PHBV. This was not the case for the PHBH laminate. It should be noted that water permeation is much less sensitive to voiding than oxygen permeation, due to the high surface tension of water.^29^ The situation for the laminates with M-MP was different. The laminates with 20% glycerol had the lowest oxygen permeance with values of ∼2 cm^3^ mm/(m^2^ day atm) and those with 25% glycerol were still slightly lower than those of the PHAs. As in the case of D-MP, the sWVTR of the M-MP laminates was, in general, lower than those of the PHAs, again showing the water barrier properties of the MPs if they are imbedded and exposed to a low water uptake.

To have a reference oxygen permeance value for a commercial polyolefin-based laminate packaging film, we have chosen here a laminate with a larger thickness. A film with an inner layer of LDPE (150 μm), an EVOH layer (44 mol % ethylene, 25 μm), and an outer layer of HDPE (600 μm) has an OP of 0.6 cm^3^ mm/(m^2^ day atm) when exposed to relative humidity on the inside of 10% and on the outside of either 65 or 75%.^44^ This is consistently lower than the OPs of the laminates in Table 3 (exposed to 50% relative humidity). The difference is 1 order of magnitude higher compared to a laminate with MP containing 25% glycerol, sandwiched between two PHBV layers (refer to the two last values in Table 3). Overall, the OTR and WVTR values presented in Table 3 are on a medium-to-high barrier level, defined according to Khalifa.^45^

## 4 Conclusions

PHAs with 19 mol % hydroxyvalerate or 10 mol % hydroxyhexanoate provided a broad melting region, making them suitable for lamination with the microbial proteins. At a lamination temperature of 110 °C, the adhesion between the semimolten PHA and the microbial protein layer was sufficient to form a three-layer semiflexible laminate, which was also possible to delaminate easily. This enables a recycling process where the rapidly biodegrading MP can be potentially used as a fertilizer and the PHA can go for mechanical recycling or industrial composting. The MP based on a mixed microbial culture showed in general superior properties compared to that based on the *D. tsuruhatensis* strain. It had a higher strain at break and a higher oxygen barrier and provided better oxygen barrier properties in the laminates. However, both MPs contributed to improved laminate water vapor barrier properties and UV blocking. The Delftia-based material yielded fewer brown-colored films. In conclusion, further development also considers biobased tie-layers as an alternative to fossil-produced adhesives if a stronger adhesion is required. The combination of biobased and biodegradable PHA and MP films in a laminate structure has the potential, with further development, to compete with today’s high-barrier multilayer packaging films, where fossil-based and nonbiodegradable EVOH is used as a gas barrier.

## Supplementary Material



## Data Availability

The data that support the findings of this study are available from the corresponding authors upon reasonable request and at DOI: 10.5281/zenodo.18402609.

## Notes

FTIR data, secondary protein structures, and images on multilayers (PDF)
