# Supplementary material for: Toward Greener Multilayer Packaging Material Solutions Based on Microbial Protein and Polyhydroxyalkanoate
Source: ACS Appl Eng Mater. 2026 Feb 5;4(2):1083–92. doi: 10.1021/acsaenm.5c01169 (PMC12956136; doi:10.1021/acsaenm.5c01169)
Supplement: Supplementary file 1 [file em5c01169_si_001.pdf]

Supporting Information

Towards Greener Multilayer Packaging Material Solutions Based on Microbial protein and Polyhydroxyalkanoate

Kiran Reddy Baddigam[1*], Elodie Guilloud[1], Anna J. Svagan[1], Bor Shin Chee[2], Buket Alkan Tas[2], Margaret Brennan Fournet[2], Kim Windey[3,4], Maria Batista[5,6], Cristiana A. V. Torres[5,6], Filomena Freitas[5,6], Mikael S. Hedenqvist[1*]

[1]Department of Fibre and Polymer Technology, Polymeric Materials Division, School of Engineering Sciences in Chemistry, Biotechnology and Health. KTH Royal Institute of Technology, Stockholm 10044, Sweden;

[2]TUS Technological University of the Shannon, Centre for Polymer Sustainability, PRISM Research Institute, Midlands Midwest, Irland

[3]Avecom nv, 9032 Wondelgem, Ghent, Belgium

[4]Valpromic nv, 9032 Wondelgem, Ghent, Belgium

[5]UCIBIO – Applied Molecular Biosciences Unit, School of Science and Technology, NOVA University Lisbon, 2829-516 Caparica, Portugal

[6]Associate Laboratory i4HB - Institute for Health and Bioeconomy, School of Science and Technology, NOVA University Lisbon, 2829-516 Caparica, Portugal

*Corresponding authors; kiranreddy.baddigam@gmail.com (K. Reddy Baddigam), mikaelhe@kth.se (M. S. Hedenqvist

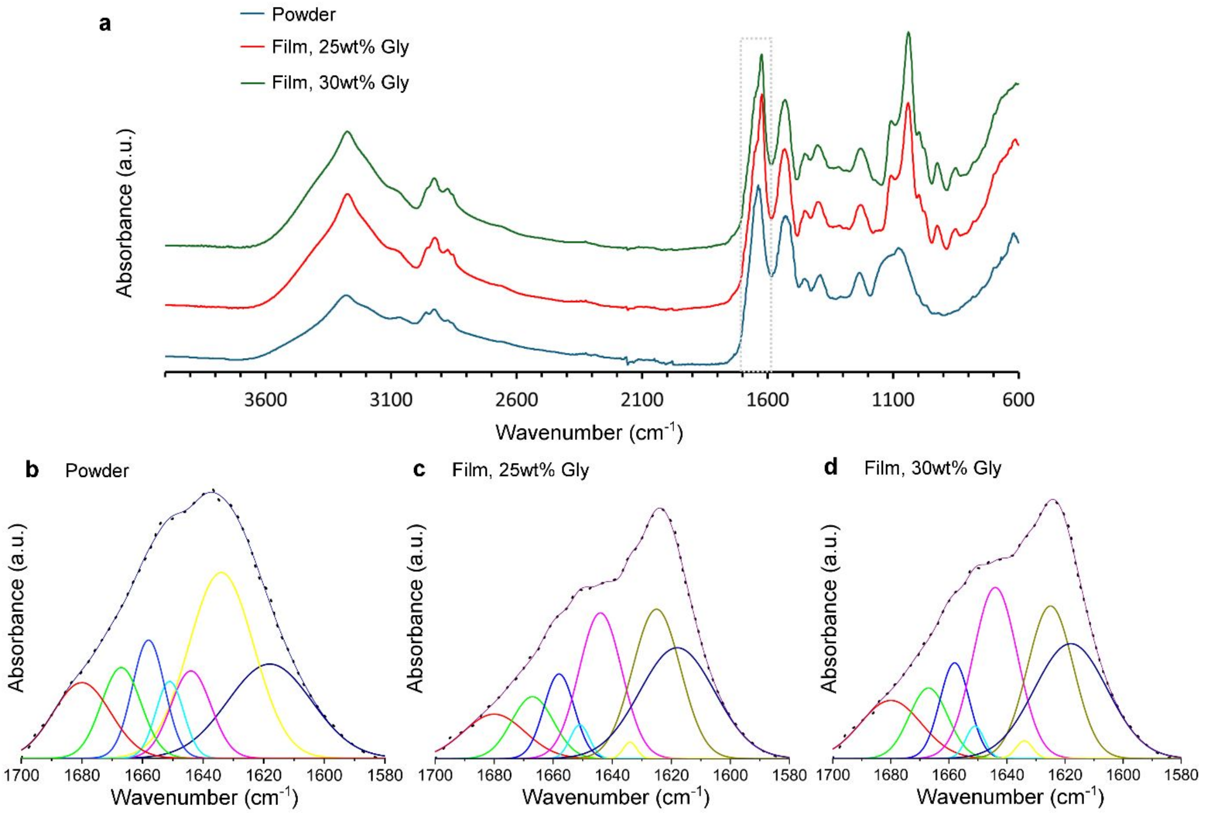

Figure S1. FTIR spectra for (a) the Delftia MB powder, two MB films containing glycerol (Gly, 25 and 30 wt% Gly). In (b-d): deconvoluted FTIR spectra in the amide I region 1580 – 1700 cm^{-1}. The dotted lines are experimental base-line corrected FTIR data.

Table S1. Secondary structures of the proteins (Cho et al.[1]) present in MB powder (Delftia) and the pressed MB films with different glycerol contents. The values within parenthesis are the uncertainties of the optimized values.

Secondary structure		Delftia, Powder	MB Film	
Glycerol content		0 wt%	25wt%	30 wt%
Position (cm^{-1})	Assignment	Relative area of gaussian components (%)		
1680	β-Sheets, weakly hydrogen-bonded peptide groups	11.6 (0.2)	9.0 (0.2)	10.8 (0.2)
1667	β-Turns	9.6 (0.3)	8.5 (0.4)	8.2 (0.3)
1658	α-Helices	10.2 (0.5)	8.4 (0.4)	8.6 (0.5)
1651	α-Helices and random coils	5.7 (0.5)	2.1 (0.4)	1.8 (0.4)
1644	Unordered	9.3 (0.5)	20.5 (1.3)	23.0 (1.4)
1634	β-Sheets, weakly hydrogen-bonded peptide groups	32.3 (1.2)	0.7 (0.2)	0.9 (0.5)
1625	β- Sheets, strongly hydrogen-bonded peptide groups	n.a.	23.5 (1.7)	21.2 (1.6)
1618	β- Sheets, strongly hydrogen-bonded peptide groups	21.2 (0.6)	27.3 (1.0)	25.4 (0.9)

n.a. = not attained

REFERENCE

(1) Cho, S. W.; Gällstedt, M.; Johansson, E.; Hedenqvist, M. S. Injection-molded nanocomposites and materials based on wheat gluten. *Int. J. Biol. Macromol.* **2011**, *48*, 146-152. doi: 10.1016/j.ijbiomac.2010.10.012.

D-MP-30/PHBH D-MP-25/PHBH M-MP-20/PHBH

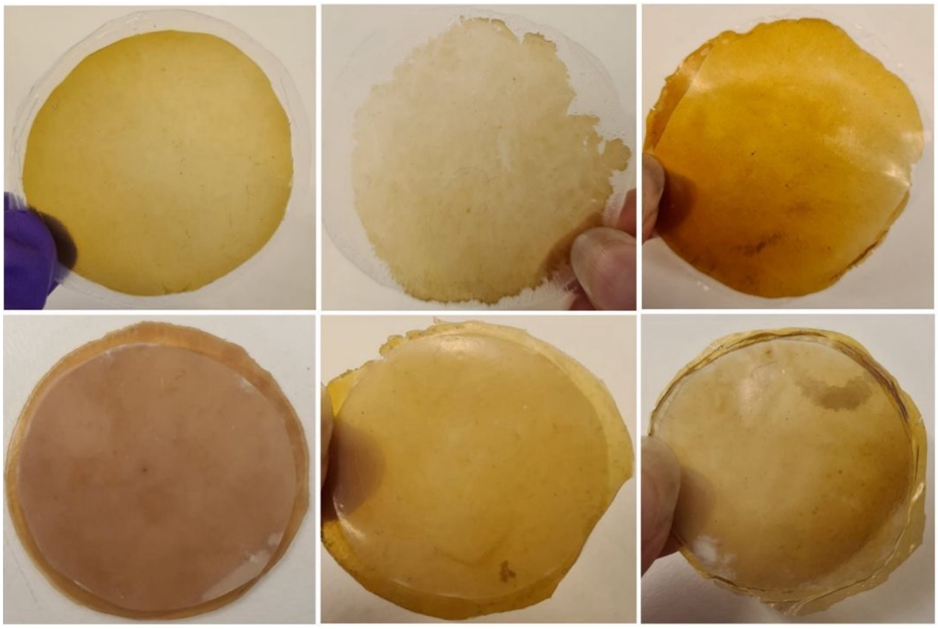

D-MP-30/PHBV D-MP-25/PHBV M-MP-20/PHBV

Figure S2. Illustrations of different 3-layer laminates.
